# Supplementary material for: Human-triggered magnification of erosion rates in European Alps since the Bronze Age
Source: Nat Commun. 2024 Feb 10;15:1246. doi: 10.1038/s41467-024-45123-3 (PMC10858959; doi:10.1038/s41467-024-45123-3)
Supplement: Supplementary file 1 — Supplementary Information [file 41467_2024_45123_MOESM1_ESM.pdf]

Nature Communications – Supplementary Information

Human-triggered magnification of erosion rates in European Alps since the Bronze Age

W. Rapuc[1,2*], C. Giguet-Covex[1], J. Bouchez[3], P. Sabatier[1], J. Gaillardet[3,4], K. Jacq[5], K. Genuite[6], J. Poulenard[1], E. Messager[1] & F. Arnaud[1]

[1]EDYTEM, CNRS, Université Savoie Mont Blanc, 73000, Chambéry, France
[2]Department of Earth Sciences, Durham University, Durham DH1 3LE, United Kingdom
[3]Institut de Physique Du Globe de Paris, Université Paris Cité, CNRS, 75005, Paris, France
[4]Institut Universitaire de France, France
[5]Laboratoire Commun SpecSolE, Envisol, CNRS, Université Savoie Mont Blanc, 73000, Chambéry, France
[6]UMR PACEA 5199, CNRS, Université de Bordeaux, 33615, Pessac, France
*email : william.rapuc@univ-smb.fr

Supplementary Notes 1. Sedimentology of Lake Bourget core sections

Sediments collected in the deep basin of Lake Bourget present two different units: an organic gyttja at the top of the sequence followed by a homogeneous grey clayey unit developing downwards. Unit I (0 - 30 cm) corresponds to an alternation of very dark grey and very light grey clayey laminae with a predominance of diatoms, authigenic calcite crystals and organic matter and is equivalent to the uppermost unit previously[1]. This unit presents the highest organic matter content, i.e., 2.5%[2] and results from the eutrophication of the lake initiated in 1933 yr CE[1,3,4]. Unit II, covers the rest of the sequence and corresponds to light grey clay composed mainly by authigenic carbonates associated with detrital particles[2,3].

Unit I present high values of Mn and low values of Ti and Ca. Conversely, in Unit II, Ti signal increases downwards and remains high until 5 m. Ca signal is high (> 500 kcps) from the bottom of the sequence until 2 m and then decrease (Supplementary Fig. 3). Apart for event layers, Mn signal is almost constant in the sediment sequence. Few upward-fining darker layers interrupt the continuous sedimentation. Two types of layers are observable:

(i) Homogenite-type deposits, with an erosive coarse base, a homogeneous dark grey silty part, and a light grey clayey top. One of these layers corresponds to a mass-wasting deposit, linked to the 1822 earthquake[5,6].

(ii) Turbidite-type deposits, with a normal upward-fining are also observable in the sediment section. These deposits are related to flood events[7,8].

Rhône river sediments only enter the lake during flood events[9], however, only major events with high sediment loading will create an identifiable layer, while low intensity floods will only participate to the continuous sediment accumulation[1]. Only the continuous sedimentation will be considered as it more probably corresponds to the erosion of the entire catchment of Lake Bourget.

Supplementary Method 1. Chronology

Nineteen samples of terrestrial macro remain were analyzed at the LMC14 laboratory (CNRS, Gif-sur-Yvette) to provide a radiocarbon ages. All samples were calibrated using IntCal20[10]. A first age-depth model was produced using the "rbacon" R-package[11] to test the reliability of each date within the model from Bayesian uncertainties. Only one ^{14}C age was considered too young and was excluded from the model (Supplementary Table 1) to avoid reversals and inconsistencies (Supplementary Fig. 4A). The R-package "clam"[12] was then used to obtain a smooth sedimentation rate (SR) for every millimetre of the sequence. Event layers that were previously described (Supplementary Fig. 3) have been interpreted as instantaneous deposits[1,8]. The sum of their depths represents 273.4 cm for the 13.71 m of the sediment sequence. To provide the best age-depth model, an event-free depth was produced by subtracting the thicknesses of event deposits from the sediment depth[13]. Historical information, such as (i) the beginning of the lake hypoxia in 1933[14], corresponding to the onset of Unit I (31.1 cm) and (ii) the 1973 homogenite (18.5 cm) observed in several sediment sequences of Lake Bourget[8], were used as chronological indicators to better constrain the model on the uppermost part of the sediment sequence.

The age-depth model was generated with the remaining 18 calibrated ages and historical information. The best fit was obtained with the R code package "clam"[12] by applying a smooth spline model with 0.4 for the smooth parameter. The sedimentation rate presented hereafter was calculated without event layers (Supplementary Fig. 4B). Finally, all instantaneous events were reintegrated to the age-depth model (Supplementary Fig. 4C) to provide a date for all event layers.

Supplementary Method 2. Estimation of the proportion of detrital silicates

To estimate the proportion of detrital silicates (PDS) in the lake, we selected the Ti signal[15] from XRF analyses, and used it as a proxy of detrital inputs in the lake[7,9,16]. The turbidite-type layer, interpreted as a flood event, with the highest Ti values (Supplementary Fig. 3) was selected as being composed at 100% of detrital sediment. This layer presents organic and carbonate contents of 7 and 9.8%, respectively (obtained from LOI results, considering LOI550° equivalent to the organic content and LOI950° equivalent to the carbonate content[17]). Considering that this event has a detrital origin, 83% of the layer corresponds to detrital silicates. The PDS in the whole sediment sequence can be obtained by diving the value of Ti over time ($[Ti]^t$) by the maximal Ti value obtained in the selected flood event ($[Ti]_{max}$). As the flood event is also composed of 17 % of detrital organic matter and carbonates, a weighting coefficient of 0.83 is applied:

$$PDS = \frac{[Ti]^t}{[Ti]_{max}} \times 0.83 \quad (1)$$

To evaluate the evolution of erosion over the Holocene in the Lake Bourget catchment, a siliciclastic flux (g.cm^{-2}.yr^{-1}) was calculated from the multiplication of SR (cm.yr^{-1}), a dry bulk density (DBD in g.cm^{-3}) and PDS:

$$SCF = SR \times DBD \times PDS \quad (2)$$

To obtain siliciclastic flux (SCF) values at high resolution, DBD model as a function of time (t) was computed using the following equation:

$$DBD_{high-res} = 3 \cdot 10^{-5t} + 0.738 \quad (3)$$

The high-resolution values of DBD from this model are in very good agreement with the measured DBD values (r^2=0.9246). The obtained SCF values are presented in Supplementary Fig. 5. According to previous studies, SCF is used here as a proxy of erosion in the Lake Bourget catchment over time[9,15]. The SCF signal presents a mean value of 0.03 ± 0.02 g.cm^{-2}.yr^{-1}, with maximum and minimum values of 0.110 and 0.011 g.cm^{-2}.yr^{-1}, respectively.

Supplementary Method 3. Geochemical analyses

Methods for neodymium isotope measurements

Isotope measurements were all performed at the PARI analytical platform of IPGP. After powder digestion, Nd was separated from the sample matrix by extraction chromatography. We used a combination of TRU-spec and Ln-spec resins for Nd[18]. Isotope ratios were measured by multi-collector inductively coupled plasma mass spectrometry (MC-ICPMS; Neptune, Thermo-Fisher Scientific). Instrumental and natural mass fractionation on the $^{143}Nd/^{144}Nd$ ratio were corrected for using an exponential law, the measured $^{146}Nd/^{144}Nd$ ratio and its natural abundance ratio, taken as 0.7218. Accuracy was checked through repeated measurements of a NIST pure-Nd, isotope reference material[19] ($^{143}Nd/^{144}Nd$ = 0.511418), with a measured 2 S.D of 2.7 x 10^{-6}, and the NIST soil reference material SRM2709a (0.512381 ± 0.000028). For convenience we report Nd isotope ratios as εNd values:

$$\varepsilon\mathrm{Nd} = \left(\frac{\left({}^{143}\mathrm{Nd}/{}^{144}\mathrm{Nd}\right)_{samples}}{\left({}^{143}\mathrm{Nd}/{}^{144}\mathrm{Nd}\right)_{CHUR}} - 1\right) \times 10\,000 \quad (4)$$

with $\left({}^{143}\mathrm{Nd}/{}^{144}\mathrm{Nd}\right)_{\mathrm{CHUR}}$ (CHUR stands for "chondritic uniform reservoir") equal[20] to 0.512638. Each sample was measured once or twice during a session, and the 95% confidence interval of the measurement was estimated at 0.4 εNd unit from repeated measurements of the NIST reference material SRM2709a at mass spectrometric signal levels similar to those of samples.

Lake sediments

In the siliciclastic fraction of Lake Bourget sediments, values of εNd range from -11.4 to -10.4 over the Holocene (Supplementary Fig. 5). As the resolution of our εNd signal is low for recent times we decided to add data from previous studies conducted on Lake Bourget sediment sequences [9,21]. We only selected data originating from sediment sequence located in the distal part of the Lake Bourget deep basin (LDB01 and B16; Supplementary Fig. 2) to avoid peculiar sub-catchment signature linked to extreme flood events from the Rhône and Arve rivers. Data from samples collected in flood deposits were also discarded. Selected data are presented in Source Data.

River sediments

River sediments taken in the Rhône and Arve sub-catchments rivers are characterized by εNd values ranging from -5.6 to -12.5. These river sediments originate from the high-altitude basins of the Mont-Blanc Massif, thus reflective of the magmatic calco-alkaline-type rock sources of the region, which tend to present the most radiogenic values. For sub-catchments draining sedimentary rocks, εNd values are between -9 and -12.5. Generally, εNd values tend to increase downstream, with lower values present in the Sallanches sub-catchment (3.A; Source Data) and highest values in southeast sub-catchments such as of Fier and Leysse rivers. The εNd values of the Rhône and Arve rivers samples range between -6.7 and -11.3, whereas their [Nd] concentrations range from 12.3 to 72.9 ppm, thereby covering the range described by their tributaries (Source Data).

Supplementary Method 4. Mixing model

Detail on the mixing model computation is provided in the Method section of the main text of this study. We highlight here that beyond accounting for analytical uncertainties, an additional sensitivity test was run to evaluate the impact of the εNd isotope signatures and Nd concentrations selected for each rock end member. This is particularly important as in order to account for the entirety of the compositional array of Lake Bourget sediments, we had to choose one particular river sediment sample (3.A, Sallanches river) as being representative of the non-glaciated (sedimentary) end member, although the composition of this sample itself might result from a mixture between detrital inputs from both sedimentary and magmatic rocks due to the presence in the selected sub-catchment of glacial deposits originating from the Mont Blanc region. Nevertheless, we note that if we were to identify a pure non-glaciated end member, it would have even lower Nd concentration and εNd values (Supplementary Fig. 6), meaning that the detrital contribution from the glaciated region would become uniformly higher for all lake sediment samples, leaving the relative trends discussed in this study unchanged.

"Geological" uncertainties of 0.8 epsilon-units on εNd, together with a 30% relative uncertainty on Nd concentrations were attributed to each end member, and an additional Monte Carlo procedure was performed in the same manner as that used for propagating analytical uncertainty (Supplementary Fig. 6). Regardless of the exact composition of the two end members within the ranges given above, the non-glaciated source remains the predominant contributor to the continuous accumulation of siliciclastic material over time in the Lake Bourget. Therefore, the results from our mixing model can be reliably used to discuss the evolution of the contribution of each source in terms of temporal variations in erosion processes in the catchment.

The results of the mixing model indicate that the non-glaciated region contributes on average to $89.1^{+3.6}_{-3.1}$ % of the total siliciclastic sedimentation in Lake Bourget, whereas the glaciated region, corresponding to magmatic rock source is the smallest contributor with a mean contribution of $10.9^{+3.1}_{-3.6}$ % (Supplementary Fig. 6; Source Data).

Supplementary Method 5. Estimation of the source region areas

To compute the sediment yield associated with each geochemical end member, it is necessary to evaluate the area of the catchment that contributes to the corresponding sediment flux. To do so, we combine all similar rock types from the BRGM geological map database (https://infoterre.brgm.fr/) by using QGIS software. For the non-glaciated end member, we aggregate all lithologies corresponding to sediment rock types, such as: conglomerates, mudstone, marlstones, calcareous marl, argillaceous limes, arenites, Quaternary deposits, and metamorphic rocks. As we only focus here on the physical erosion of silicate rocks, carbonate rocks such as limestone and dolostone are not considered in the estimation of the non-glaciated end member area, nor in the total surface area. For the glaciated end member, we combine granite and pegmatite rock types of the Mont Blanc region. The area covered by each rock type (*i.e.*, granite and sedimentary rocks) represent 3,804 and 158 km² for "Sedimentary rocks" (*i.e.*, non-glaciated region) and "Mont Blanc" (*i.e.*, glaciated region) regions, respectively. Obtained SY_i values (Method) are presented in the Source Data. $SY_{Glaciated}$ presents a mean value two-times higher than $SY_{Non-glaciated}$.

Supplementary Method 6. Correlation and breakpoints between the erosion rates of glaciated and non-glaciated regions

Correlation between the erosion signal of the glaciated and the non-glaciated regions may be used to compute a theoretical signal of erosion for the non-glaciated region considering only the effects of climate. Thanks to the computation of several rolling correlation curves (see Method), a mean correlation signal through time was extracted. Three main breakpoints were identified at 6,437, 5,584 and 3,858 yr cal BP on this signal (Supplementary Fig. 7). They represent the major shifts into the correlation between glaciated and non-glaciated regions erosion. Except for the period between 6,437 and 5,584 yr cal BP, the erosion of the non-glaciated and glaciated regions displays a very good correlation (r= 0.86, p-value = 10^{-7}). The lack of correlation between 6,437 and 5,584 yr cal BP can be explained by significant waning of glaciers at high altitudes in the European Alps reported for this period[22], thus modifying the relationship between the erosion of glaciated and non-glaciated regions.

From this correlation it is possible to obtain a relationship between the erosion signal of both glaciated and non-glaciated regions for the beginning of the Holocene:

$$SY_{Non-glaciated\ area} = 0.1474 * SY_{glaciated\ area} + 0.5461 \quad (5)$$

The best fit between the erosion rates of the non-glaciated and glaciated regions was obtained (Supplementary Table 2) using a 5th-order polynomial (r = 0.89), but for the sake of simplicity we instead use a linear relationship (r = 0.86). This equation can be applied to the rest of the erosion record, allowing us to obtain an expected erosion signal for the non-glaciated region (Supplementary Fig. 7).

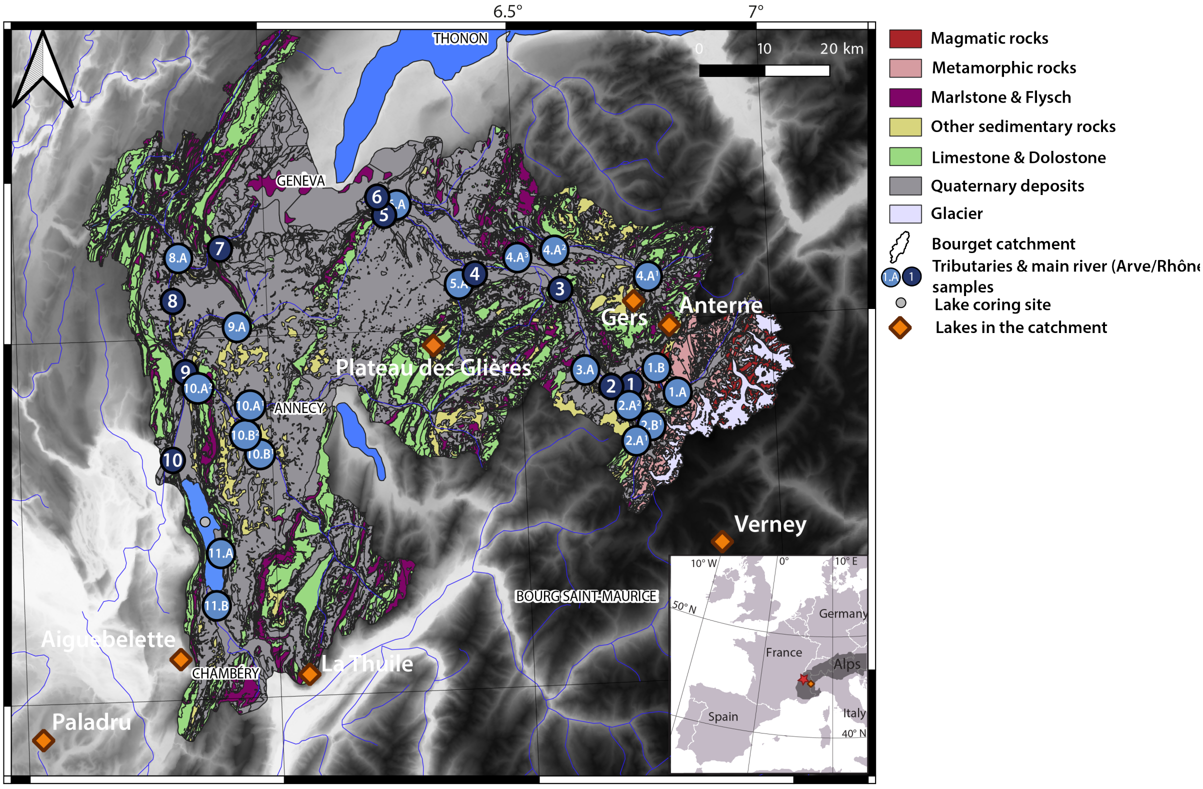

Supplementary Figure 1 - Map of the study area, and location of sampling sites. The geological data was obtained from BRGM in 2019 (https://infoterre.brgm.fr/), and is superimposed on a shaded relief map (grey shading). The locations of all river sediment sampling sites are reported as blue circles, and those of Lake Aiguebelette, Anterne, Gers, La Thuile, Paladru, and Verney are reported as orange diamonds).

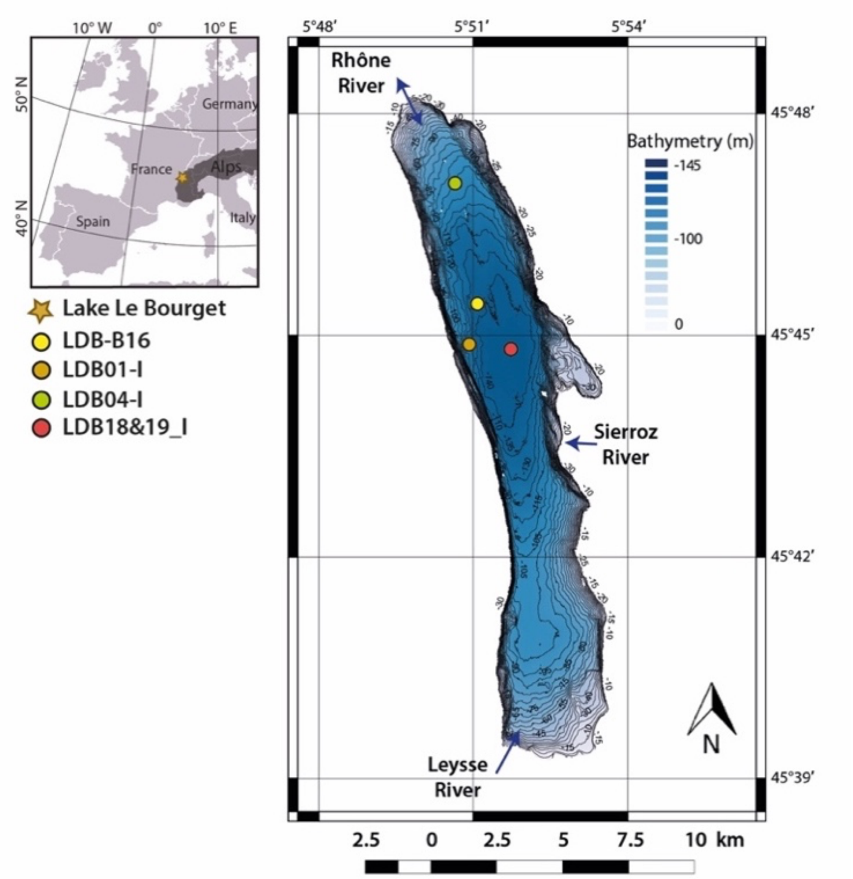

Supplementary Figure 2 – Bathymetric map of the Lake Bourget. With the location of each of the two sediment sequences described and used in this study. Bathymetric map derived from previously published data[23].

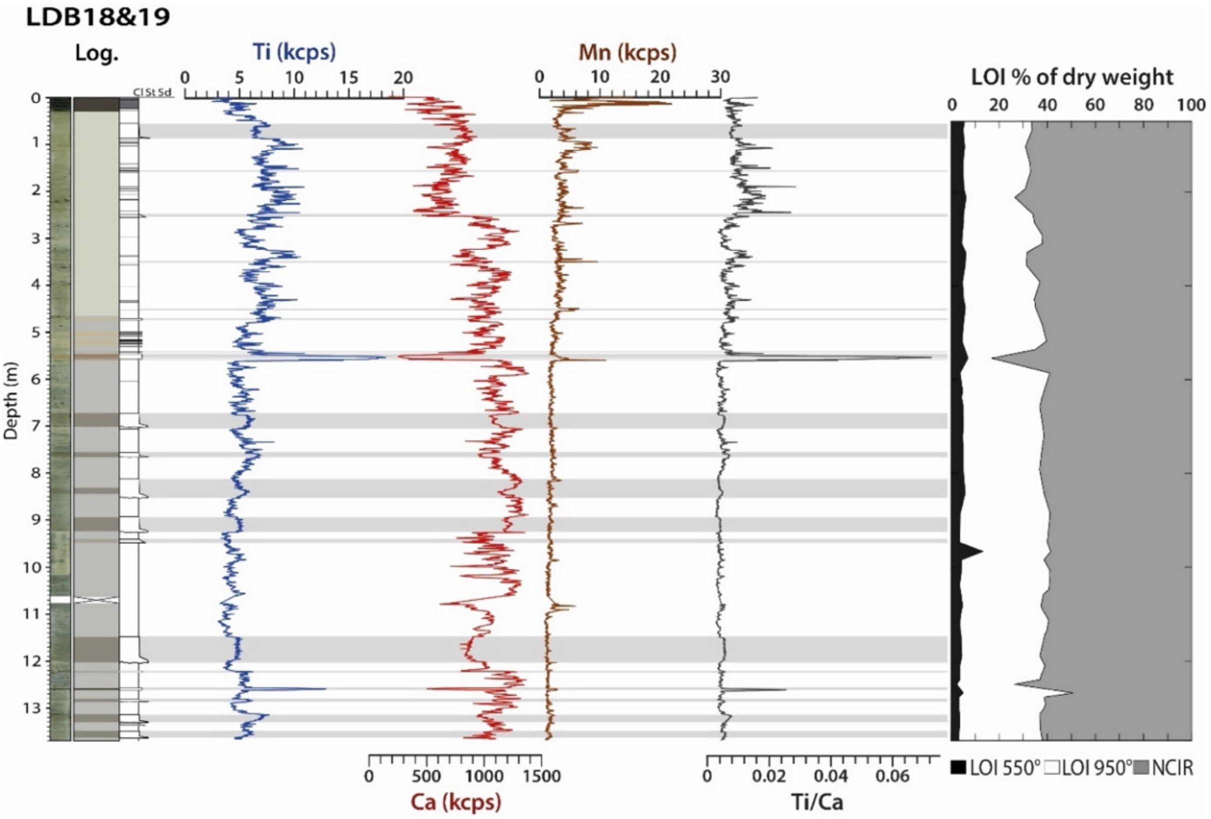

Supplementary Figure 3 - Main sedimentological and geochemical results from the Lake Bourget sediment sequence. A picture of the sediment sequence is associated with the lithological description, geochemical results: Ti, Ca, Mn contents expressed in kilo counts per second (kcps) and Ti/Ca signal; and loss on ignition (LOI) expressed in %. NCIR stands for noncarbonated residue. Source data are provided as a Source Data file.

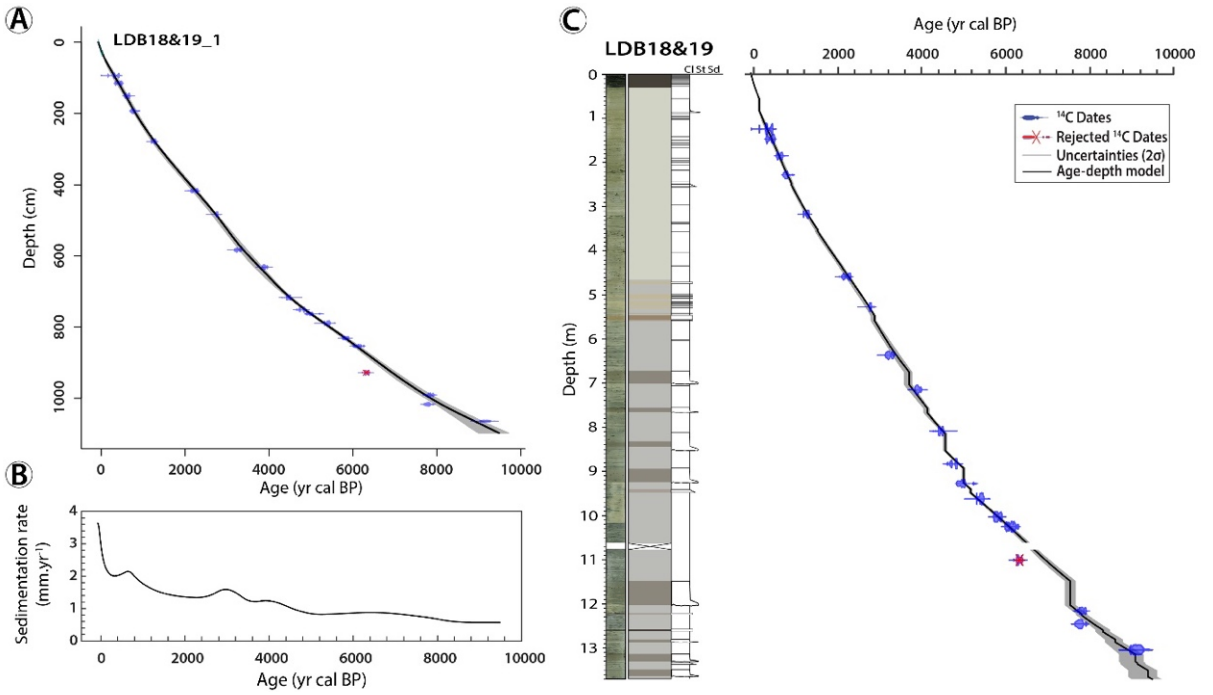

Supplementary Figure 4 – Lake Bourget sediment cores LDB18&19 age-depth models. (A) Age–depth model associating radiocarbon and historical event dates as a function of depth (note that parts of the core corresponding to short accumulation events were removed) and (B) corresponding sedimentation rate. (C) Complete age-depth model. Source data are provided as a Source Data file.

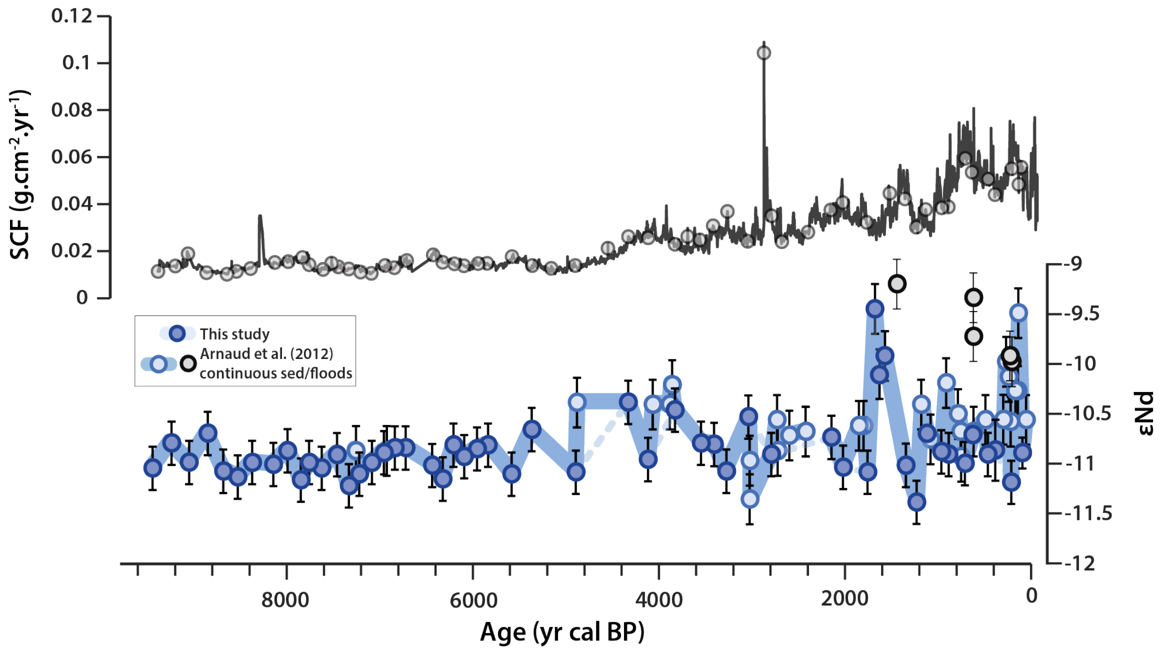

Supplementary Figure 5 – Silicate detrital accumulation rate and neodymium isotopic composition of Lake Bourget sediment over the Holocene. Silicate detrital sedimentation rate (SCF, for siliciclastic flux) and Nd isotope ratios (expressed as εNd) of the Lake Bourget over the last 9,500 years obtained from the LDB18&19 lake sediment sequence. The siliciclastic flux was obtained from XRF Ti signal, age-depth modelling, and density measurements (Supplementary Method 2). Error bars on the εNd curves represent the 95% confidence intervals corresponding to analytical uncertainty. Source Data are provided as a Source Data file.

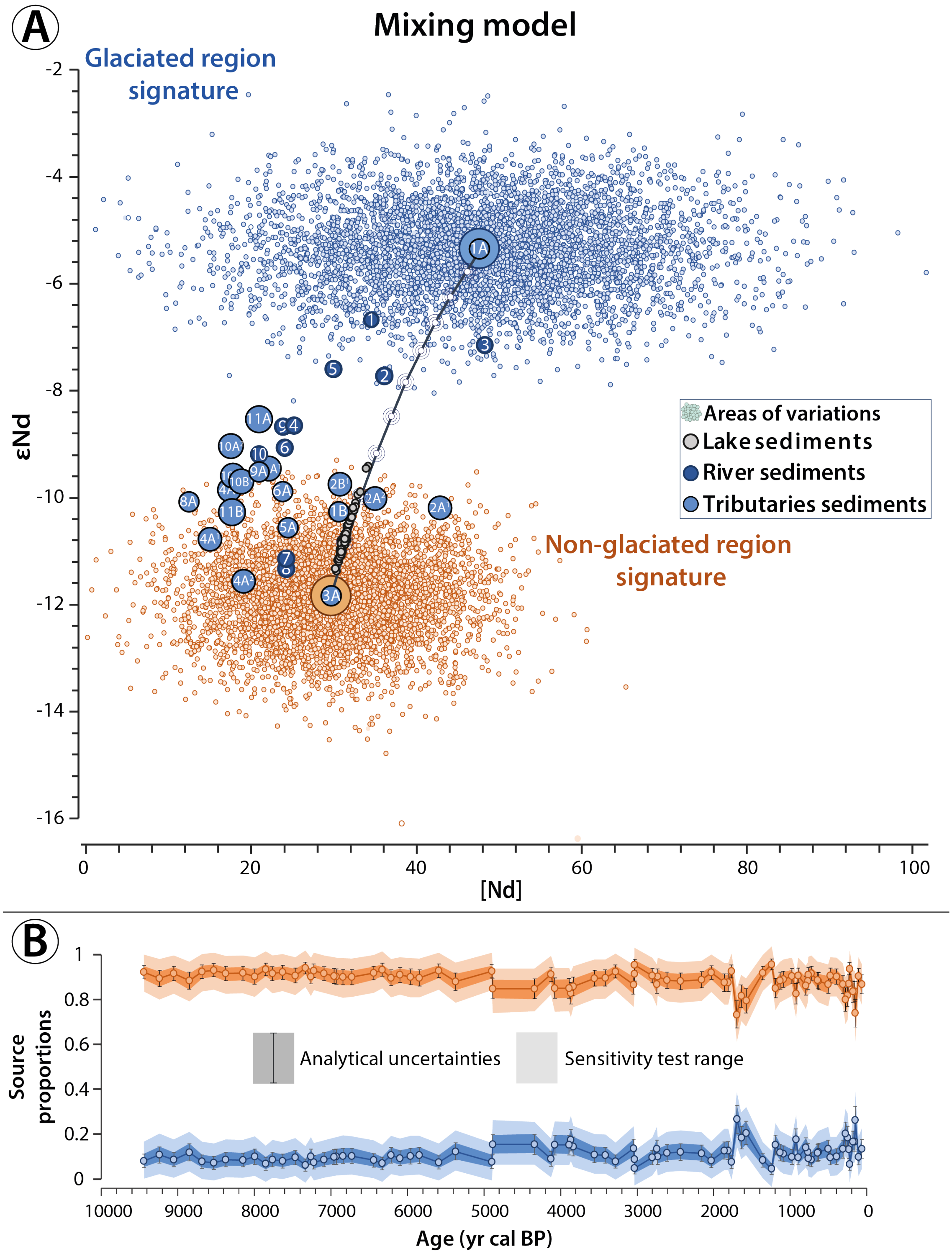

Supplementary Figure 6 – Results from the geochemical mixing model. (A) Nd isotope signatures (expressed as εNd) of the two identified rock sources, used as end members for explaining the composition of lake sediments. Samples 3.A and 1.A are used to constrain the composition of the rock end members. Lake sediments are shown as grey circles within an array described by the mixing hyperbola. The blue and orange symbols correspond to the composition of each Monte Carlo iteration used for the sensitivity test. (B) Fractional contributions of each rock source to the Lake Bourget sediment against time (yr cal BP). The propagation of analytical uncertainties is represented as error bars and by dark shaded areas, corresponding to D84 and D16 of the distribution of the parameters obtained from Monte Carlo simulations (5,000 draws; see Methods). The light shaded areas represent the propagation of the uncertainty associated with the composition of the three rock sources, again with D84 and D16 of the distribution of the parameters obtained from Monte Carlo simulation. Source data are provided as a Source Data file.

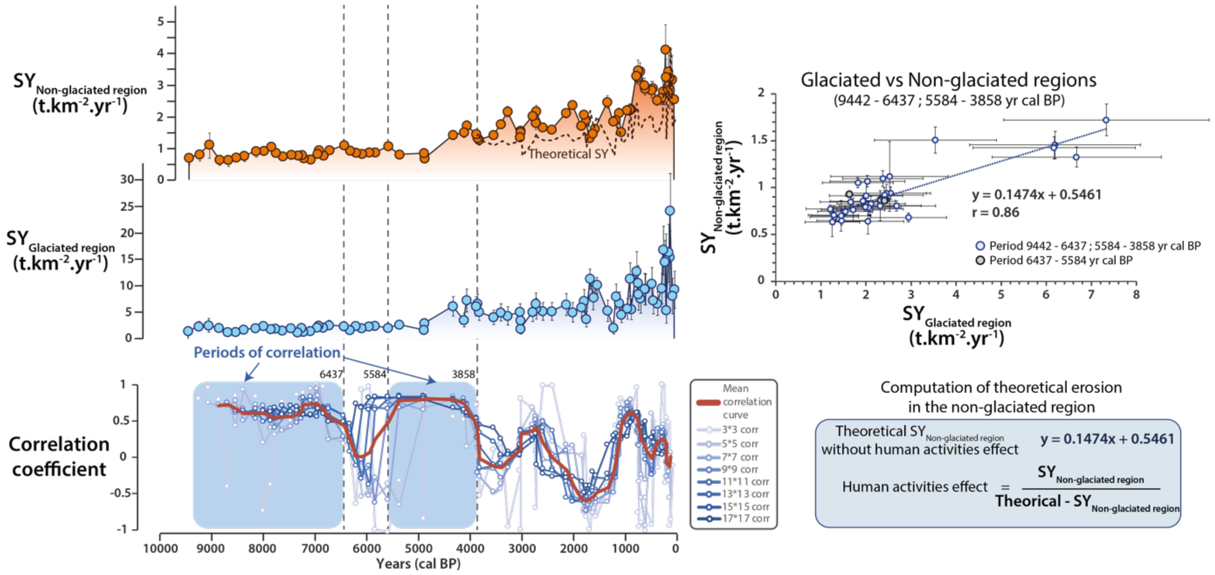

Supplementary Figure 7 – Computation of the effect of Human activities on erosion. Erosion signals from non-glaciated and glaciated regions of the Lake Bourget catchment, and roll correlation curves obtained with different widths, as a function of time. The error bars correspond to the standard deviation obtained using a Monte Carlo approach (Method). A biplot of the erosion signal of the two different regions of the catchment allows us to observe the relationship between the erosion rate of the two regions between 9,442 and 6,437 yr cal BP, and between 5,584 and 3,858. The best fit between these two erosion signals over this period was obtained using a 5th-order polynomial (r = 0.89), but for the sake of simplicity we instead use a linear relationship (r = 0.86). The linear best-fit equation can then be applied to the rest of the erosion record, allowing us to calculate an expected erosion signal for the non-glaciated region. This expected erosion signal is represented with a dotted line on the upper left. Source data are provided as a Source Data file.

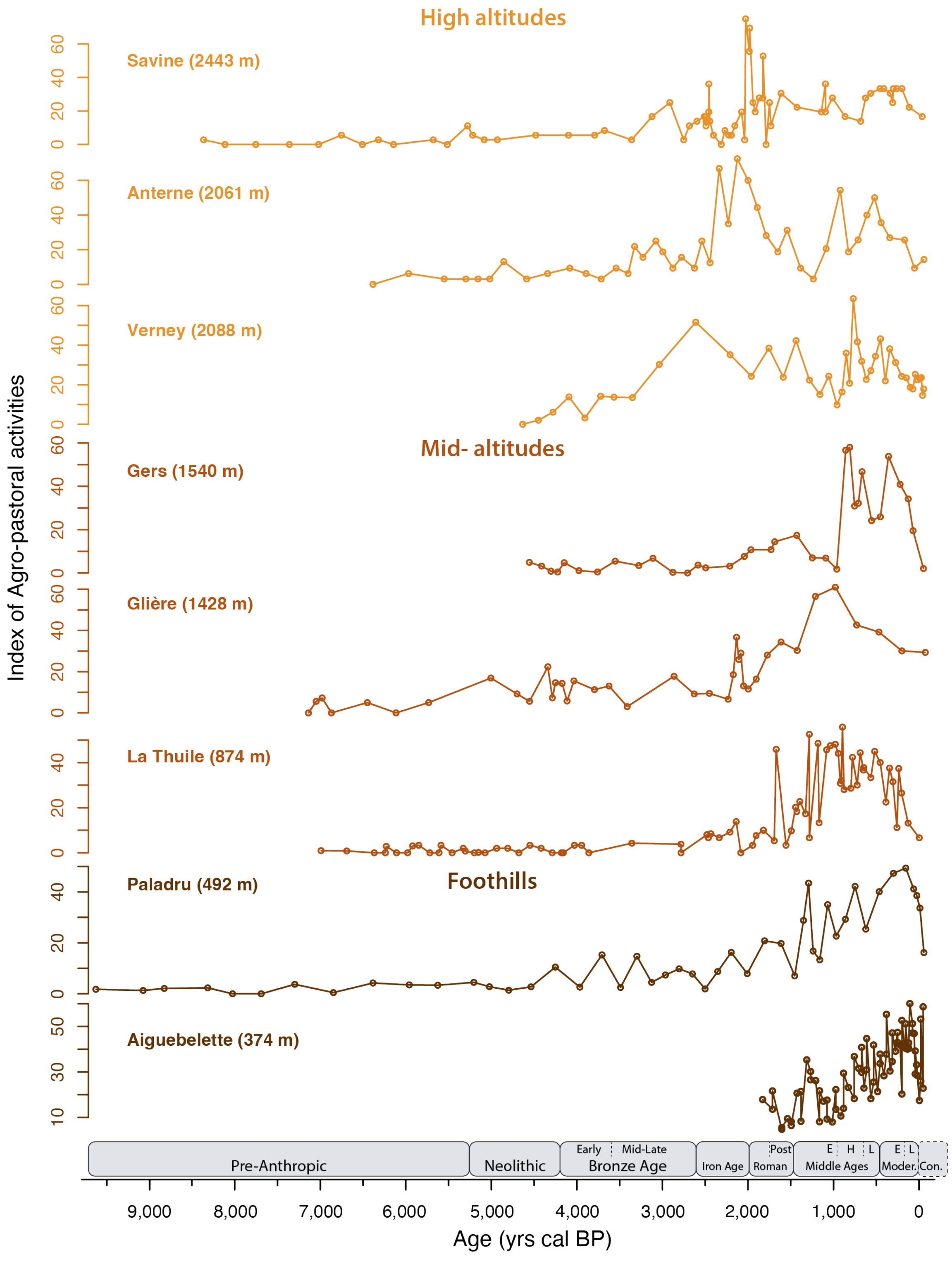

Supplementary Figure 8 – Index of agro-pastoral activities per sites in the western European Alps. Data used to compute the indexes presented here derived from previous studies conducted on lake sediments sequences retrieved in the western European Alps. Information about lake locations, catchments and taxa used are detailed in Supplementary Table 3. Details on the method used to compute these indexes and on the data origin are given in a previous study[24]. Source data are provided as a Source Data file.

Supplementary Table 1 - Radiocarbon ages for the LDB18&19 sediment sequence. The composite depth was calculated by excluding the thicknesses of each event deposits that were considered as instantaneous deposits. The sample in bold corresponds to the date excluded from the age-depth model.

Sample name	Core	MCD (cm)	Composite depth (cm)	Radiocarbon age	Age cal yr BP (2 σ range)	Type
Sac 57152	LDB18_I_A01A	124	93.3	265 ± 30	-2-431	Wood
Poz-118211	LDB18-A-01A	146.5	115.1	375 ± 30	318-503	Wood
Poz-117956	LDB18-B-01	183	150.8	670 ± 50	552-685	Wood
Poz-117957	LDB18-B-01	227	192.9	845 ± 30	689-892	Wood
Sac 57151	LDB18_I_A02A	316.9	278.4	1335 ± 30	1185-1303	Wood
Poz-117958	LDB18-B-02A	459.45	417	2180 ± 30	2119-2309	Wood
Sac 57153	LDB18_I_E03A	526.5	483.7	2630 ± 30	3166-3344	Wood
Poz-117968	LDB18-B-03A	637.5	583.5	3040 ± 30	3262-3469	Wood
Sac 57154	LDB18_I_C_02A	714	631.5	3585 ± 30	3831-3976	Wood
Poz-117955	LDB18-C-02A	809.75	717.1	3975 ± 30	4305-4525	Wood
Sac 57150	LDB18_I_D02A	882.5	751.5	4235 ± 30	4653-4858	Wood
Poz-117969	LDB18-D-02A	927	762.8	4410 ± 30	4867-5256	Wood
Sac 57149	LDB18_I_D02A	961.5	788.9	4650 ± 30	5312-5465	Wood
Poz-117861	LDB19-I-B-02A	1003.8	831.2	5075 ± 35	5744-5908	Wood
Poz-117862	LDB19-I-B-02B	1025.8	853.2	5345 ± 35	6002-6267	Wood
**Poz-117948**	**LDB19-I-B-03A**	**1100.6**	**928**	**5520 ± 40**	**6223-6402**	**Wood**
Poz-117949	LDB19-I-B-03A	1217.6	990.6	7000 ±40	7737-7934	Wood
Poz-117950	LDB19-I-A-10	1246.7	1017	6960 ± 40	7691-7922	Wood
Poz-117951	LDB19-I-A-10	1303.95	1064.3	8200 ± 50	9017-9296	Wood

Supplementary Table 2 – Statistical test between erosion signals from glaciated and non-glaciated regions. Results of the statistical test between the two variables ($SY_{Non\text{-}glaciated\ region}$ and $SY_{Glaciated\ region}$) between 9442 and 6437 and 5584 and 3858 yr cal BP.

	Variable 1	*Variable 2*
Mean	2.619	0.932
Variance	2.715	0.081
Observations	31	31
**Pearson's product-moment coefficient**	**0.856**	
Hypothetical difference of means	0	
Degrees of freedom	30	
T - value	6.649	
**P(T<=t) unilateral**	**1E-07**	
T critical value (unilateral)	1.697	
P(T<=t) bilatéral	2E-07	
T critical value (bilateral)	2.042	

Supplementary Table 3 – Location and information about all the lakes located close to Lake Bourget and used to reconstruct past human activities.

Areas	Foothills		Mid-altitude			High-altitude		
Lakes	Paladru	Aiguebelette	La Thuile	Gers	Plateau des Glières	Verney	Anterne	Savine
**Lake information**								
Altitude (m a.s.l)	492	374	874	1540	1428	2088	2061	2443
Lat	45°27'3.02"N	45°33'11.56"N	45°31′50.63″N	46° 1'36.93"N	45°57'55.89"N	45°41′19.8″N	45°59'27.51"N	45°10'30.71"N
Long	5°31'44.30"E	5°48'0.09"E	6°3′39.9″E	6°43'46.13"E	6°19'59.65"E	6°52′57.06″E	6°47'52.55"E	6°54'47.77"E
Area (km2)	3.73	5.5	0.06	0.05	0.005	0.2	0.12	0.09
Depth max. (m)	36	71	8	5.6	0.5	21	13.2	7
Type	Lake	Lake	Lake	Lake	Peat Bog	Lake	Lake	Lake
Origin	Glacial	Glacial	Glacial	Glacial	?	Glacial	Glacial	Glacial
Distance to Lake Bourget (km)	41.95	21.41	28.11	74.19	43.77	79.56	77.21	103.58
Reference	[25]	[26]	[17]	[27]	[28]	[29]	[30]	[31]
**Catchment information**								
Area (km2)	55	58.9	1.6	5	0.1075	3.6	2.55	3.5
Geological settings	Molasse and fluvial deposits	Limestone and marls	Limestone and marls	Taveyannaz sandstones	Flysch and Marls	Limestones and sandstones	Shales, calcshists and limestones	Gneiss and mica schist
Altitude min. (m a.s.l)	492	374	874	1540	1428	2088	2061	2443
Altitude max. (m a.s.l)	750	1441	1209	2385	1612	2900	2494	3310
Altitude mean. (m a.s.l)	621	907.5	1041.5	1962.5	1520	2494	2277.5	2876.5
Vegetation belts	Hilly & Montane belt	Hilly & Montane belt	Montane belt	Subalpine belt	Subalpine belt	Subalpine/alpine belt	Subalpine/alpine belt	Alpine/Nival belt
References	[25]	[26]	[17]	[27]	[28]	[29]	[30]	[31]
**Previous Scientific studies**								
Paleo-environments	Pollen	Pollen + DNA	Pollen + DNA	Pollen + DNA	Pollen + DNA	Pollen + DNA	DNA	DNA
references	[25]	[26]	[17]	[27]	Original Data	[32,33]	[34,35]	[33]
Archeology	Lake-dwelling	Lake-dwelling	x	x	x	[36,37]	[38]	x
references	[39]	?			x		x	x
Paleo-climatology	x	x	x	Flood chronicle	x	x	Flood chronicle	Flood chronicle
references	x	x	x	[27]	x	x	[30]	[31]
Erosion rates	Sedimentation rate & Ti(XRF)	Loss On Ignition (LOI)	Sedimentation rate & Ti(XRF)	Sediment Yield	x	Sedimentation rate & LOI(NCIR)	Sedimentation rate & Flood frequency	Sedimentation rate
references	[25]	[26]	[17]	[27]	x	[29]	[40]	[31]
**Taxa included in the indexes of agro-pastoral activities**	Plantago lanceolata and major/media, Rumex sp, Urticaceae, Cerealia, Secale, Fagopyrum sp., Cannabis sativa/Humulus lupulus, Vitis sp., Juglans sp., and Castanea sp. from pollen	Plantago sp., Rumex sp, Urtica sp., Avena sp., Fagopyrum sp., Humulus, Cannabis sativa, Vicia faba, Pisum, Phaseolus, Apium graveolens, Beta vulgaris, Cynara cardunculus, Viticeae, Juglandaceae, Castanea, Prunus, Maleae, Sus scrofa, Bos taurus from sedaDNA & Plantago lanceolata and major/media, Rumex-type, Urtica sp., Chenopodiaceae, Cerealia, Secale, Fagopyrum sp., Cannabis sativa/Humulus lupulus, Vitis sp., Juglans sp., Castanea sp. and Prunus sp. from pollen	Plantago and Rumex sp, Cerealia and Cannabis sativa/Humulus lupulus from pollen, and Bos, Ovis sp., Vitaceae, Juglandaceae, Pyrus sp., Prunus sp. and Vicia faba from sedaDNA	Sporormiella spores and Plantago sp., Rumex sp., Bos, Ovis sp. and Capra hircus from sedaDNA	Sporormiella spores and Plantago sp., Rumex-type, Urticaceae, Chenopodiaceae from pollen	Sporormiella spores and Plantago sp., Rumex, Bos and Ovis sp. from sedaDNA	Plantago sp., Bos and Ovis sp. from sedaDNA	Plantago sp., Bos and Ovis sp. from sedaDNA

Supplementary References

1. Giguet-Covex, C. *et al.* Sedimentological and geochemical records of past trophic state and hypolimnetic anoxia in large, hard-water Lake Bourget, French Alps. *J. Paleolimnol.* **43**, 171–190 (2010).
2. Millet, L. *et al.* Reconstruction of the recent history of a large deep prealpine lake (Lake Bourget, France) using subfossil chironomids, diatoms, and organic matter analysis: towards the definition of a lake-specific reference state. *J. Paleolimnol.* **44**, 963–978 (2010).
3. Debret, M. *et al.* North western Alps Holocene paleohydrology recorded by flooding activity in Lake Le Bourget, France. *Quat. Sci. Rev.* **29**, 2185–2200 (2010).
4. Jenny, J.-P. *et al.* A spatiotemporal investigation of varved sediments highlights the dynamics of hypolimnetic hypoxia in a large hard-water lake over the last 150 years. *Limnol. Oceanogr.* **58**, 1395–1408 (2013).
5. Beck, C. "Late Quaternary lacustrine paleo-seismic archives in north-western Alps: Examples of earthquake-origin assessment of sedimentary disturbances". *Earth-Sci. Rev.* **96**, 327–344 (2009).
6. Chapron, E., Beck, C., Pourchet, M. & Deconinck, J.-F. 1822 earthquake-triggered homogenite in Lake Le Bourget (NW Alps). *Terra Nova* **11**, 86–92 (1999).
7. Rapuc, W. *et al.* XRF and hyperspectral analyses as an automatic way to detect flood events in sediment cores. *Sediment. Geol.* **409**, 105776 (2020).
8. Jenny, J.-P. *et al.* A 4D sedimentological approach to reconstructing the flood frequency and intensity of the Rhône River (Lake Bourget, NW European Alps). *J. Paleolimnol.* **51**, 469–483 (2014).
9. Arnaud, F. *et al.* Lake Bourget regional erosion patterns reconstruction reveals Holocene NW European Alps soil evolution and paleohydrology. *Quat. Sci. Rev.* **51**, 81–92 (2012).
10. Reimer, P. J. *et al.* The IntCal20 Northern Hemisphere Radiocarbon Age Calibration Curve (0–55 cal kBP). *Radiocarbon* **62**, 725–757 (2020).
11. Blaauw, M. *et al.* rbacon: Age-Depth Modelling using Bayesian Statistics. (2021).
12. Blaauw, M. Methods and code for 'classical'age-modelling of radiocarbon sequences. *Quat. Geochronol.* **5**, 512–518 (2010).
13. Arnaud, F. *et al.* Flood and earthquake disturbance of 210Pb geochronology (Lake Anterne, NW Alps). *Terra Nova* **14**, 225–232 (2002).
14. Jenny, J.-P. *et al.* Inherited hypoxia: A new challenge for reoligotrophicated lakes under global warming. *Glob. Biogeochem. Cycles* **28**, 1413–1423 (2014).
15. Arnaud, F. & Révillon, S. A Geochemical Approach to Improve Radiocarbon-Based Age-Depth Models in Non-laminated Sediment Series. in *Micro-XRF Studies of Sediment Cores: Applications of a non-destructive tool for the environmental sciences* (eds. Croudace, I. W. & Rothwell, R. G.) 459–472 (Springer Netherlands, 2015). doi:10.1007/978-94-017-9849-5_18.
16. Arnaud, F. *et al.* Erosion under climate and human pressures: An alpine lake sediment perspective. *Quat. Sci. Rev.* (2016) doi:10.1016/j.quascirev.2016.09.018.
17. Bajard, M. *et al.* Erosion record in Lake La Thuile sediments (Prealps, France): Evidence of montane landscape dynamics throughout the Holocene. *The Holocene* **26**, 350–364 (2016).
18. Cogez, A. *et al.* Constraints on the role of tectonic and climate on erosion revealed by two time series analysis of marine cores around New Zealand. *Earth Planet. Sci. Lett.* **410**, 174–185 (2015).
19. Caro, G., Bourdon, B., Birck, J.-L. & Moorbath, S. High-precision 142Nd/144Nd measurements in terrestrial rocks: Constraints on the early differentiation of the Earth's mantle. *Geochim. Cosmochim. Acta* **70**, 164–191 (2006).
20. Jacobsen, S. B. & Wasserburg, G. J. Sm-Nd isotopic evolution of chondrites. *Earth Planet. Sci. Lett.* **50**, 139–155 (1980).
21. Revel-Rolland, M. *et al.* Sr and Nd isotopes as tracers of clastic sources in Lake Le Bourget sediment (NW Alps, France) during the Little Ice Age: Palaeohydrology implications. *Chem. Geol.* **224**, 183–200 (2005).
22. Ivy-Ochs, S. *et al.* Latest Pleistocene and Holocene glacier variations in the European Alps. *Quat. Sci. Rev.* **28**, 2137–2149 (2009).
23. Ledoux, G., Lajeunesse, P., Chapron, E. & St-Onge, G. Multibeam Bathymetry Investigations of Mass Movements in Lake Le Bourget (NW Alps, France) Using a Portable Platform. in *Submarine Mass Movements and Their Consequences* (eds. Mosher, D. C. et al.) 423–434 (Springer Netherlands, 2010). doi:10.1007/978-90-481-3071-9_35.
24. Giguet-Covex, C. *et al.* Long-term trajectories of mountain agro-ecosystems in the North-Western Alps. *Reg. Environ. Change* **23**, 58 (2023).
25. Simonneau, A. *et al.* Holocene land-use evolution and associated soil erosion in the French Prealps inferred from Lake Paladru sediments and archaeological evidences. *J. Archaeol. Sci.* **40**, 1636–1645 (2013).
26. Messager, E. *et al.* Two Millennia of Complexity and Variability in a Perialpine Socioecological System (Savoie, France): The Contribution of Palynology and sedaDNA Analysis. *Front. Ecol. Evol.* **10**, (2022).
27. Bajard, M. *et al.* Pastoralism increased vulnerability of a subalpine catchment to flood hazard through changing soil properties. *Palaeogeogr. Palaeoclimatol. Palaeoecol.* **538**, 109462 (2020).
28. Julien, A., Doyen, E., Giguet-Covex, C. & Messager, E. 7000 ans d'histoire de la végétation et des activités agro-pastorales sur le plateau des Glières (Alpes du Nord françaises). in *Actes du Colloque final du projet PCR 'Ecosystèmes montagnards'* Accepted (Presses Universitaires de Grenoble, 2023).
29. Bajard, M. *et al.* Progressive and regressive soil evolution phases in the Anthropocene. *Catena* **150**, 39–52 (2017).
30. Giguet-Covex, C. *et al.* Frequency and intensity of high-altitude floods over the last 3.5 ka in northwestern French

Alps (Lake Anterne). *Quat. Res.* **77**, 12–22 (2012).
31. Sabatier, P. *et al.* 6-kyr record of flood frequency and intensity in the western Mediterranean Alps – Interplay of solar and temperature forcing. *Quat. Sci. Rev.* **170**, 121–135 (2017).
32. Bajard, M. *et al.* Long-term changes in alpine pedogenetic processes: Effect of millennial agro-pastoralism activities (French-Italian Alps). *Geoderma* **306**, 217–236 (2017).
33. Chen, W. Quantitative approaches to the analysis of sedimentary DNA to understand past biodiversity and ecosystem functioning. (Université Grenoble Alpes, 2019).
34. Giguet-Covex, C. *et al.* Long livestock farming history and human landscape shaping revealed by lake sediment DNA. *Nat. Commun.* **5**, 1–7 (2014).
35. Pansu, J. *et al.* Reconstructing long-term human impacts on plant communities: an ecological approach based on lake sediment DNA. *Mol. Ecol.* **24**, 1485–1498 (2015).
36. Rey, P.-J. & Moulin, B. L'occupation des versants du col du Petit-Saint-Bernard au Néolithique: premier bilan des données acquises lors des campagnes de sondages du programme Alpis Graia. *Collect. EDYTEM Cah. Géographie* **L'homme dans les Alpes, de la pierre au métal**, 241–255 (2018).
37. Crogiez-Pétrequin, S. Alpis Graia (col du Petit-Saint-Bernard, Savoie et Val d'Aoste) : découvertes anciennes et recherches récentes. *Gall. Archéologie Gaules* **73**, 113–118 (2016).
38. Rey, P.-J. *et al.* De la préhistoire aux temps modernes dans les alpages d'Anterne et Pormenaz. Une approche de l'occupation de la moyenne montagne entre les Fiz et les Aiguilles Rouges. Résultats du programme de sondages. in *Archéologie en milieu de montagne dans la région Auvergne-Rhône-Alpes. Actes de la table ronde de Clermont-Ferrand, 6 décembre 2019* 55–93 (Presses universitaires Blaise Pascal, 2022).
39. Brochier, J.-L., Borel, J.-L. & Druart, J.-C. Les variations paléoenvironnementales de 1000 avant à 1000 après J.C. et la question des « optima » climatiques de l'Antiquité tardive et du Moyen Âge sur le piémont des Alpes du nord à Colletière, lac de Paladru, France. *Quat. Rev. Assoc. Fr. Pour Létude Quat.* 253–270 (2007) doi:10.4000/quaternaire.1125.
40. Giguet-Covex, C. *et al.* Changes in erosion patterns during the Holocene in a currently treeless subalpine catchment inferred from lake sediment geochemistry (Lake Anterne, 2063 m a.s.l., NW French Alps): The role of climate and human activities. *The Holocene* **21**, 651–665 (2011).
