# Supplementary material for: Human-triggered magnification of erosion rates in European Alps since the Bronze Age
Source: Nat Commun. 2024 Feb 10;15:1246. doi: 10.1038/s41467-024-45123-3 (PMC10858959; doi:10.1038/s41467-024-45123-3)
Supplement: Supplementary file 3 — Reporting Summary [file 41467_2024_45123_MOESM3_ESM.pdf]

nature portfolio

Corresponding author(s): William Rapuc
Last updated by author(s): Nov 3, 2023

Reporting Summary

Nature Portfolio wishes to improve the reproducibility of the work that we publish. This form provides structure for consistency and transparency in reporting. For further information on Nature Portfolio policies, see our Editorial Policies and the Editorial Policy Checklist.

Statistics

For all statistical analyses, confirm that the following items are present in the figure legend, table legend, main text, or Methods section.

n/a	Confirmed	
☐	☒	The exact sample size (n) for each experimental group/condition, given as a discrete number and unit of measurement
☐	☒	A statement on whether measurements were taken from distinct samples or whether the same sample was measured repeatedly
☐	☒	The statistical test(s) used AND whether they are one- or two-sided<br>*Only common tests should be described solely by name; describe more complex techniques in the Methods section.*
☒	☐	A description of all covariates tested
☒	☐	A description of any assumptions or corrections, such as tests of normality and adjustment for multiple comparisons
☐	☒	A full description of the statistical parameters including central tendency (e.g. means) or other basic estimates (e.g. regression coefficient) AND variation (e.g. standard deviation) or associated estimates of uncertainty (e.g. confidence intervals)
☒	☐	For null hypothesis testing, the test statistic (e.g. F, t, r) with confidence intervals, effect sizes, degrees of freedom and P value noted<br>*Give P values as exact values whenever suitable.*
☒	☐	For Bayesian analysis, information on the choice of priors and Markov chain Monte Carlo settings
☒	☐	For hierarchical and complex designs, identification of the appropriate level for tests and full reporting of outcomes
☒	☐	Estimates of effect sizes (e.g. Cohen's d, Pearson's r), indicating how they were calculated

Our web collection on statistics for biologists contains articles on many of the points above.

Software and code

Policy information about availability of computer code

Data collection	No software was used for data collection. Whenever data from previous studies were used, this was specified in the data tables along with the origin of the data.
Data analysis	The age-depth model of the sediment section was performed with “rbacon” and “clam” packages on R version 4.2.1 .<br>Box plots were realised using “ggplot2” and “grid” packages and “geom_boxplot” function on R version 4.2.1<br>Rolling correlations were computed thanks to the "zoo" package and the "rollapply" function on R version 4.2.1<br>The sliding correlation curves (roll correlations) were averaged and extracted using Matlab software<br>The Monte Carlo method used to compute uncertainties in the fractional contributions was executed using a Box–Muller transform on Excel software.

For manuscripts utilizing custom algorithms or software that are central to the research but not yet described in published literature, software must be made available to editors and reviewers. We strongly encourage code deposition in a community repository (e.g. GitHub). See the Nature Portfolio guidelines for submitting code & software for further information.

Data

Policy information about availability of data

All manuscripts must include a data availability statement. This statement should provide the following information, where applicable:

- Accession codes, unique identifiers, or web links for publicly available datasets
- A description of any restrictions on data availability
- For clinical datasets or third party data, please ensure that the statement adheres to our policy

The authors declare that the data supporting the findings of this study are available within the paper and its supplementary information files.

Research involving human participants, their data, or biological material

Policy information about studies with human participants or human data. See also policy information about sex, gender (identity/presentation), and sexual orientation and race, ethnicity and racism.

Reporting on sex and gender	*Use the terms sex (biological attribute) and gender (shaped by social and cultural circumstances) carefully in order to avoid confusing both terms. Indicate if findings apply to only one sex or gender; describe whether sex and gender were considered in study design; whether sex and/or gender was determined based on self-reporting or assigned and methods used. Provide in the source data disaggregated sex and gender data, where this information has been collected, and if consent has been obtained for sharing of individual-level data; provide overall numbers in this Reporting Summary. Please state if this information has not been collected. Report sex- and gender-based analyses where performed, justify reasons for lack of sex- and gender-based analysis.*
Reporting on race, ethnicity, or other socially relevant groupings	*Please specify the socially constructed or socially relevant categorization variable(s) used in your manuscript and explain why they were used. Please note that such variables should not be used as proxies for other socially constructed/relevant variables (for example, race or ethnicity should not be used as a proxy for socioeconomic status). Provide clear definitions of the relevant terms used, how they were provided (by the participants/respondents, the researchers, or third parties), and the method(s) used to classify people into the different categories (e.g. self-report, census or administrative data, social media data, etc.) Please provide details about how you controlled for confounding variables in your analyses.*
Population characteristics	*Describe the covariate-relevant population characteristics of the human research participants (e.g. age, genotypic information, past and current diagnosis and treatment categories). If you filled out the behavioural & social sciences study design questions and have nothing to add here, write "See above."*
Recruitment	*Describe how participants were recruited. Outline any potential self-selection bias or other biases that may be present and how these are likely to impact results.*
Ethics oversight	*Identify the organization(s) that approved the study protocol.*

Note that full information on the approval of the study protocol must also be provided in the manuscript.

Field-specific reporting

Please select the one below that is the best fit for your research. If you are not sure, read the appropriate sections before making your selection.

☐ Life sciences ☐ Behavioural & social sciences ☒ Ecological, evolutionary & environmental sciences

For a reference copy of the document with all sections, see nature.com/documents/nr-reporting-summary-flat.pdf

Ecological, evolutionary & environmental sciences study design

All studies must disclose on these points even when the disclosure is negative.

Study description	Here, we reconstruct the quantitative effect of climate fluctuations and human activities on erosion rates in Lake Bourget catchment (western European Alps) based on a multi-proxy, source-to-sink approach conducted on both river and lake sediments and relying on neodymium isotopic geochemistry. We also used plant sedaDNA and pollen from 8 lake sediment sequences to reconstruct a regional index of agro-pastoral activities.
Research sample	The study of the evolution of the erosion in the catchment was mainly conducted on samples from a sediment sequence retrieved from Lake Bourget depocenter and on river sediment samples. Sediment sections were split, photographed at high-resolution (20 pixels.mm−1), described and logged in detail. Identification of specific layers in the overlapping sections combined with correlations of XRF-core scanner signals allowed the creation of a 13.7-m-long sediment sequence called "LDB18&19" (IGSN n°TOAE0000000005 & n° TOAE0000000006). To characterize the source of sediments accumulated in the Lake Bourget deep basin, 29 river sediment samples were collected from flood deposits in all sub-catchments directly contributing to the Arve and Rhône rivers.
Sampling strategy	In the sediment sequence, fifty-nine 2 cm-thick sediment samples were collected using graduated syringes for geochemical analyses. A constant sampling step of 10 cm was applied where no event deposit interrupted the continuous sedimentation. For periods when

the resolution of our study was too low, we used data from previous studies also carried out on the sediments of Lake Bourget. For river sediment samples, sub-catchments covered by this sampling set represent the entire catchment area of the Lake Bourget. Samples were collected manually, and only the fraction finer than 63 µm was used, to ensure that river and lake sediments were comparable.

Data collection

William Rapuc, Pierre Sabatier and Fabien Arnaud carried out the fieldwork thanks to a UWITEC platform (EDYTEM/LSCE/C2FN) with the help of staff from the DT-INSU CLIMCOR and several researchers and students from the EDYTEM laboratory. William Rapuc labelled, described and analysed the lake and river sediment samples. William Rapuc produced the age-depth model and the chemistry in a clean lab to obtain data on elemental concentration and isotopic composition. Julien Bouchez and William Rapuc carried out the MC-ICP-MS and ICP-QMS measurements with the help of Pierre Burckel. Charline Giguet-Covex collected the data and created the agro-pastoral activity indices by altitudinal ranges. Kim Genuite and William Rapuc used GIS data to obtain the surface areas covered by each type of rock in the catchment area.

Timing and spatial scale

Two missions were required to collect the sediment cores from Lake Bourget, which were carried out between June 2018 and June 2019. The river sediment samples were taken between January and February 2018. All the analyses were then carried out between June 2018 and October 2020.

Data exclusions

Only one 14C age was considered too young and was excluded from the model. All data and samples, including the one that has been removed are included in the raw data presented in Supplementary Information

Reproducibility

All the details for this work to be reproduced are detailed in the manuscript, and replica and standard measurements were also carried out where possible to check the quality and reproducibility of the measurements.

Randomization

Randomization was not relevant as no experiment was conducted.

Blinding

Blinding was not relevant in our study because for data collection as we have only used methods that have already been published elsewhere and are regularly used in the community.

Did the study involve field work? ☒ Yes ☐ No

Field work, collection and transport

Field conditions

Lake Bourget is a peri-alpine lake located in the western European Alps (France) at 231.5 m a. s. l. All the other studied sites are located within the catchment of Lake Bourget or nearby. The catchment covers a large area (4,976 km2) stretching from the sub-alpine plains (230 m a.s.l) to the outer Crystalin massif and the Mont Blanc glacier region (4,808 m a.s.l). The region is influenced by an alpine climate: winters are cold and very wet, with heavy precipitation in the form of rain or snow, while summers are hot, heavy and stormy.

Location

The sediment sequence of Lake Bourget called “LDB18&19” (IGSN n°TOAE0000000005 & n° TOAE0000000006) is 13.7-m-long and was retrieved precisely by 45°44.717'N; 5°51.789'E. The precise location of river sediment samples is given in Supplementary Information and does not exceed the limits of the catchment of Lake Bourget.

Access & import/export

The sampling sites (lake and rivers) were accessible by car. Once the samples had been collected, they were all transported and stored in the EDYTEM laboratory's cold room.

Disturbance

No environmental disturbance has been caused.

Reporting for specific materials, systems and methods

We require information from authors about some types of materials, experimental systems and methods used in many studies. Here, indicate whether each material, system or method listed is relevant to your study. If you are not sure if a list item applies to your research, read the appropriate section before selecting a response.

Materials & experimental systems

n/a	Involved in the study
☒	☐ Antibodies
☒	☐ Eukaryotic cell lines
☒	☐ Palaeontology and archaeology
☒	☐ Animals and other organisms
☒	☐ Clinical data
☒	☐ Dual use research of concern
☒	☐ Plants

Methods

n/a	Involved in the study
☒	☐ ChIP-seq
☒	☐ Flow cytometry
☒	☐ MRI-based neuroimaging
